# Comparing Cannabis Use Across Diagnosed Conditions: Apples and Oranges?

**DOI:** 10.1093/geroni/igab046.1965

**Published:** 2021-12-17

**Authors:** Brian Kaskie

**Affiliations:** University of Iowa, Iowa City, Iowa, United States

## Abstract

Although researchers have identified medications that relieve symptoms of Multiple Sclerosis (MS), none are entirely effective and some persons with multiple sclerosis (PwMS) use alternatives. Our study compared cannabis use among PwMS (N=135) and persons diagnosed with arthritis (N=582) or cancer (N=622) who participated in the Illinois medical cannabis program. We tested for significant differences across psychological well-being, quality of life and three behavioral outcomes, and also considered effects of co-occurring prescription opioid use. A majority of all individuals used cannabis to address pain and improve quality of sleep. PwMS reported lower levels of productivity, exercise and social activity, and cannabis was less helpful with improving these particular outcomes. Most persons used cannabis for sleep or digestive problems and we found no differences across groups in terms of well-being and quality of life. This comparative evaluation suggests cannabis mechanisms are not specific as much as they impact common processes.

